# Finishing protocols of orthodontic attachments determine surface roughness and susceptibility to microbial colonization in vitro

**DOI:** 10.1038/s41598-026-46360-w

**Published:** 2026-04-02

**Authors:** Jorge Bazileu Miranda Mota, Isabella Barbosa dos Santos Justino, João Victor Frazão Câmara, Karla Lorene de França Leite, Mônica Tirre de Souza Araújo

**Affiliations:** 1 School of Dentistry, Federal University of Rio de Janeiro, Rio de Janeiro, RJ Brazil; 2 Saarland University, Homburg, Germany

**Keywords:** Orthodontic appliances, Dental Plaque, Orthodontics, Biofilms, Diseases, Health care, Medical research, Microbiology

## Abstract

To evaluate, in vitro, the microbiological contamination and surface roughness of orthodontic attachments finished using different excess-removal procedures. Thirty composite resin attachments were fabricated and allocated into three groups (n = 10/group): G1- no removal of excess resin; G2—excess removed using a No. 15 scalpel blade; and G3—excess removed using a 24-blade low-speed bur (Orthometric). All specimens underwent non-contact 3D profilometry to determine baseline surface roughness (Sa). Samples were then exposed for 24 h at 37 °C under microaerophilic conditions to a mixed inoculum (5 × 10^5^ CFU/mL) consisting of *Streptococcus mutans* ATCC 25175, *Lactobacillus casei* ATCC 393 and *Candida albicans* ATCC 90028. Post-exposure assessments included biofilm acidogenicity, CFU quantification, and repeat 3D profilometry. There were no significant differences in pH values among G1, G2, and G3. CFU counts differed significantly among groups, with the highest microbial load in G1 (709.70 ± 221.8), followed by G2 (342.65 ± 84.8) and G3 (78.3 ± 38.7) (*p* < 0.05). Initial surface roughness showed similarity between G2 and G3, both significantly smoother than G1 (*p* < 0.05). After biofilm formation, G3 exhibited the lowest Sa values, G2 showed intermediate values, and G1 remained the roughest; all groups differed significantly (*p* < 0.05). Orthodontic attachment finishing protocols significantly affects surface roughness and subsequent microbial accumulation. Leaving excess resin increases roughness and biofilm formation, whereas finishing with a 24-blade low-speed bur produces smoother surfaces and minimizes microbial contamination.

## Introduction

The increasing use of clear aligner therapy in contemporary orthodontics has led to the widespread application of composite resin attachments to enhance mechanical retention and enable complex tooth movements^1,2^. These attachments are bonded directly to enamel and frequently require removal of excess resin after placement to achieve proper contour and adaptation^3^.

In clinical practice, excess composite resin around attachments is a common occurrence. If not adequately finished, residual resin may create surface irregularities and retentive niches that favor plaque accumulation^4^. Surface roughness is a well-established determinant of microbial adhesion, as rougher surfaces increase bacterial retention and facilitate early biofilm formation^5,6^. This relationship has been extensively demonstrated for restorative dental materials, where alterations in surface morphology significantly influence biofilm development and microbial colonization^7^.

Although the importance of surface characteristics in bacterial adhesion is well documented, limited attention has been given to the finishing protocols specifically used for aligner composite attachments. In daily orthodontic practice, clinicians employ different techniques to remove excess resin, most commonly using scalpel blades or rotary instruments (e.g., multi-blade carbide burs). However, there is currently no standardized, evidence-based protocol guiding the selection of these techniques.

Increased surface irregularities are clinically relevant because rougher surfaces facilitate microbial adhesion and biofilm formation, potentially disrupting the oral microbial ecosystem and contributing to pathological conditions such as gingivitis, periodontitis, and enamel demineralization^8,9^. Despite the high clinical frequency of excess resin removal during attachment bonding, the impact of different finishing approaches on surface morphology and subsequent bacterial adhesion remains under-investigated.

Therefore, the present in vitro study aims to compare two commonly used excess resin removal techniques—scalpel blade and 24-blade low-speed bur—and to evaluate their effects on (1) surface roughness, (2) microbial colonization, and (3) the acidogenic potential (pH profile) of the resulting biofilm. By addressing the lack of standardized finishing guidelines, this study seeks to provide clinically relevant evidence to support decision-making in aligner attachment finishing procedures.

## Material and methods

### Study design

The present study was an in vitro experimental investigation conducted at the Multidisciplinary Research Laboratory of the School of Dentistry, Federal University of Rio de Janeiro (FO/UFRJ). The primary experimental units consisted of composite resin orthodontic attachments fabricated specifically for laboratory analysis. Surface roughness and microbiological contamination were evaluated using these standardized attachment specimens. For scanning electron microscopy (SEM) analysis, however, the attachments were bonded onto bovine enamel specimens to better simulate clinical substrate conditions during morphological evaluation.

**Fig. 1:**
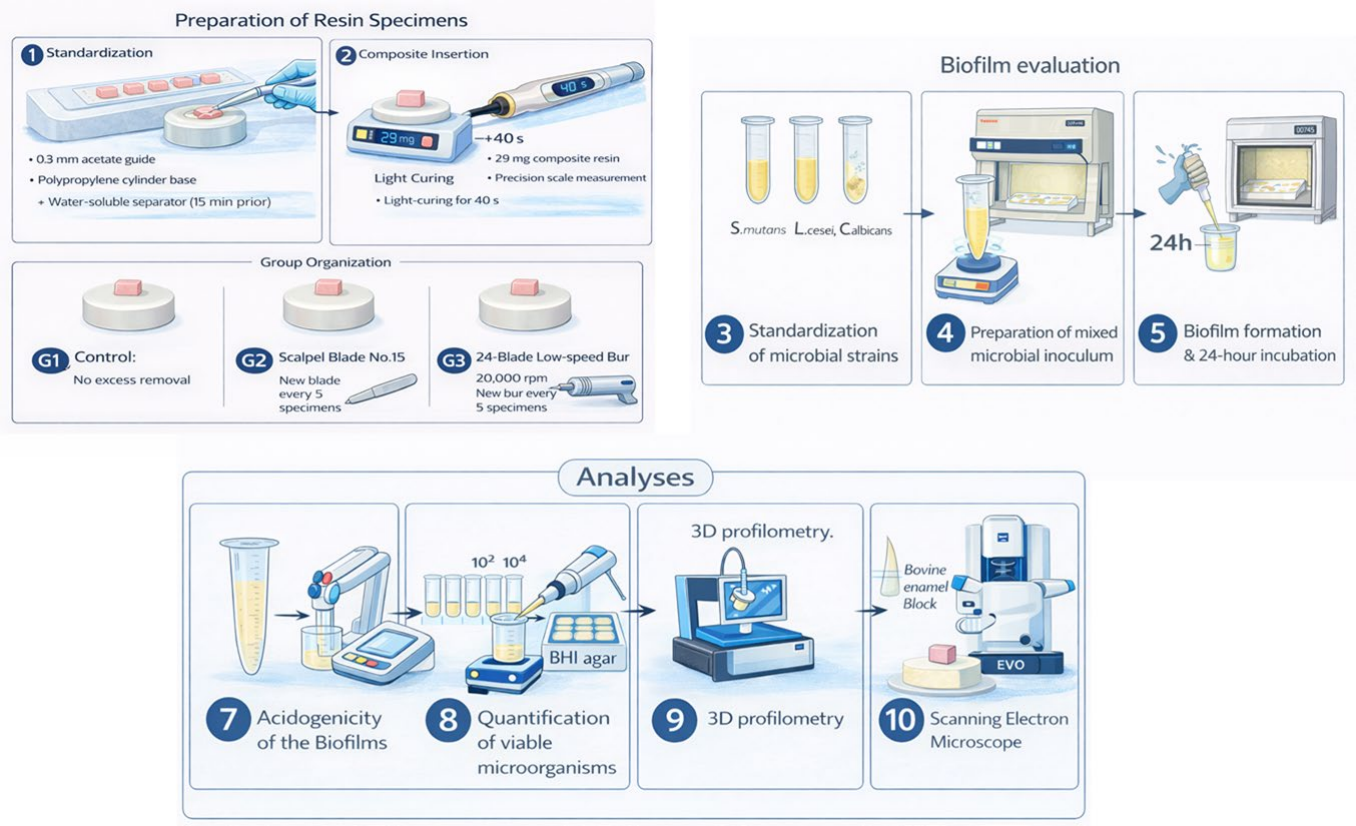
Flowchart of the study design and experimental procedures.

A total of 30 resin composite attachments were produced for this study. No formal sample size calculation was performed; therefore, a convenience sample was adopted. The specimens were randomly distributed into three experimental groups (n = 10/group) according to the excess resin removal protocol. All attachments were fabricated using Z100 resin (3 M ESPE, St. Paul, MN, USA). The control group received no excess removal. In the second group, excess material was removed with a number fifteen scalpel blade, whereas in the third group, removal was performed using a low-speed twenty-four-blade bur (Orthometric, Marília, SP, Brazil) (Fig. 1).

### Preparation of attachments and templates

For fabrication of the acetate guides, attachments were digitally designed using MeshMixer software (Autodesk, San Rafael, CA, USA), version 3.5.474. A rectangular attachment measuring 2 × 2 × 3 mm was created. A cylinder with 30 mm in diameter was positioned beneath the rectangular structure to reproduce the specimen base geometry. After completion of the virtual 3D model, the template was printed using a stereolithographic 3D printer (Anycubic Photon Mono 4 K, Shenzhen, Guangdong, China).

The printed template was used to thermoform 0.3-mm-thick acetate sheets (Bio-Art, São Carlos, SP, Brazil) in a vacuum forming machine (Plastivac P7, Bio-Art, São Carlos, SP, Brazil). Standardization of the thermoforming bubble was performed by positioning it 2.3 mm from the acetate sheet base (1.8 mm from the forming platform and 0.5 mm from the parallel indicator). This procedure ensured uniform sheet thickness and accurate reproduction of attachment morphology, particularly at the edges. One acetate guide was used for each specimen^10^.

### Pilot study for standardization of resin volume

A preliminary pilot study was conducted to determine the composite resin mass capable of producing excess material similar to that observed clinically during attachment bonding procedures. Different resin masses (25 mg, 27 mg, 29 mg, and 31 mg) were tested using the same acetate guides and bonding protocol described in the main study. The criterion for selection was the amount that consistently produced marginal resin overflow beyond the attachment template without causing complete filling deficiency or excessive material accumulation.

The 29 mg mass was selected as it consistently produced marginal resin overflow comparable to clinical conditions observed during clear aligner attachment bonding. The pilot study was conducted exclusively to standardize the experimental model and was not included in the statistical analysis of the main outcomes. This amount was weighed using a high-precision analytical scale and inserted into the attachment niche of the acetate guide.

### Bonding procedure

To standardize attachment shape, thickness, and positioning, the 0.3-mm-thick acetate guide was used during bonding. Prior to resin insertion, the guides were coated with a thin layer of water-soluble separating medium 15 min in advance, allowing adequate drying time and preventing contamination.

The guide containing the pre-weighed composite resin was positioned on a polypropylene surface with standardized smoothness conditions, serving as the base for specimen preparation. Light curing was performed for 40 s according to the manufacturer’s recommendations for the applied material thickness. After polymerization, the acetate guide was carefully removed, and excess composite resin was subjected to the removal protocols described below.

### Removal of excess resin

Removal of excess resin procedures were performed by a single calibrated operator (JBMM). Prior to the experiment, operator calibration was performed through preparation of ten preliminary specimens until consistent removal patterns were achieved.

In G2, excess composite was removed using a No. 15 scalpel blade, which was replaced after every five specimens. In G3, removal was carried out using 24-blade low-speed bur (Orthometric, Marília, SP, Brazil), also replaced after every five specimens and operated at 20.000 rpm under air cooling, without water irrigation^11^.

Assessment of complete excess removal was performed under standardized illumination using a 55 W halogen reflector light positioned at a fixed distance of 30 cm from the specimen. Complete removal was defined as the absence of visible composite flashes at the attachment margins under direct illumination, combined with the absence of detectable irregularities upon tactile examination using a sharp dental explorer. Magnification was not employed, as the protocol was designed to simulate routine clinical conditions in which attachment finishing is typically performed without optical aids.

### Surface roughness

After excess removal (G2 and G3), all specimens were rinsed with an air–water spray for 5 s to eliminate surface debris. Subsequently, the specimens were carefully detached from the resin attachments for the subsequent stages of the study. The surface roughness of the different attachments was analyzed using non-contact 3D optical profilometry (Nanovea PS50 Optical, NANOVEA Inc., Irvine, USA), prior and after the microbiological test. In this way, it was possible to characterize and surface roughness by measuring the height profiles, provided by the different methods of removing excess resin. The average value of three of volumetric roughness (Sa) (250 μm^2^), both pre- and post- experiment, was obtained for each specimen.

### Biofilm evaluation

To evaluate the retention of biofilm on attachments subjected to different multi- species, a microbiological experiment was carried out. Before the test, the specimens were placed in a 12-well polystyrene culture plate (model K12-024, Kasvi) and were sterilized in ultraviolet light (40W) in a laminar flow hood, with an exposure time of 1 h.

The strains of Streptococcus mutans (ATCC 25175), Lactobacillus casei (ATCC 393), and Candida albicans (ATCC 90028) were reactivated using standardized individual inocula prepared according to CLSI (Clinical and Laboratory Standards Institute, 2012) guidelines^12^. Aliquots of each standardized inoculum were then combined in equal proportions (1:1:1) to obtain a mixed multispecies inoculum. The strains were transferred to BHI broth supplemented with 2% sucrose (pH 7.1). The microbial suspensions were adjusted according to optical density values ranging from 0.08 to 0.2, considering wavelengths of 625 nm for bacteria and 530 nm for fungi.

Before biofilm formation, the specimens were immersed in 2 mL of artificial saliva at 37 °C for 1 h in a microbiological oven to allow the formation of an acquired salivary pellicle. The artificial saliva used in this study consisted of 1% carboxymethylcellulose, 0.0084% sodium chloride, 0.12% potassium chloride, 0.0342% monobasic potassium phosphate, 0.0146% calcium chloride, and 0.0052% magnesium chloride. After the immersion, the artificial saliva was carefully removed using a sterile pipette.

Subsequently, 2 mL of the mixed inoculum (final concentration of 5 × 10^5^ CFU/mL) containing 2% sucrose (pH 7.1) was added to each well, and the specimens were incubated at 37 °C for 24 h. The 24 h incubation period was intentionally selected to evaluate early-stage biofilm formation and initial acidogenic activity associated with different surface finishing protocols, rather than mature biofilm development. After 24 h, the culture medium from each specimen was collected for subsequent pH assessment.

The acidogenicity of the biofilms was assessed by measuring the pH of the culture medium, carried out in duplicate using a microelectrode (PHOX, Colombo, Brazil). After 24 h, each specimen was placed in an Eppendorf containing 1 mL of saline solution (NaCl). Sequentially, they were vortexed for 1 min and aliquots of suspended biofilm were used for quantification of viable microorganisms. Then, the biofilm suspension was diluted serially (10^1^ to 10^6^), in which 50 μl were collected from dilutions 10^4^ and 10^6^ and seeded on BHI agar and incubated at 37 °C for 24 h.

### Scanning electron microscope (SEM)

Qualitative surface analysis of the resin attachments was performed using Scanning Electron Microscopy (JSM-5410LV, JEOL, Tokyo, Japan) operating at 10 kV. Electron micrographs were obtained at 500× magnification. Only for the SEM analysis, sound bovine incisors were used as substrate. These specimens were not subjected to the microbiological test, since this analysis aimed exclusively to qualitatively evaluate surface morphology.

Sound bovine incisors (without cracks or structural defects, verified under a stereomicroscope) were selected and stored in 0.1% thymol solution under refrigeration (4 °C) until use. Each tooth was sectioned parallel to its long axis using a high-precision cutting machine (Isomet, Buehler, Lake Bluff, IL, USA) to obtain enamel strips. These strips were further sectioned to produce enamel blocks measuring 4 × 4 × 2 mm. The enamel surfaces were polished in a metallographic polishing machine (APL4, Arotec, Cotia, SP, Brazil) using 400-, 600-, and 1200-grit silicon carbide papers under water cooling.

After polishing, the enamel surface was etched with 37% phosphoric acid for 30 s, rinsed, and gently air-dried. A layer of adhesive system (Adper Single Bond, 3 M ESPE) was actively applied using a disposable applicator (KG Sorensen, Cotia, SP, Brazil), air-thinned for 5 s, and light-cured for 10 s according to the manufacturer’s instructions.

Subsequently, the transfer guide containing the resin attachment was positioned onto the enamel surface, and the bonding protocol was performed according to the experimental group. After fabrication, the specimens (n = 2/group) were sanitized, dehydrated when necessary, mounted on metallic stubs, and sputter-coated with a thin layer of gold prior to SEM examination.

### Statistical analysis

The data obtained from the analysis of surface roughness, acidogenicity and CFU count were organized in a database and submitted to the Statistical Package for Social Science software (version 17.0, SPSS Inc., Chicago, Illinois, USA). Data distribution was assessed using the Shapiro Wilk test (5% significance level). The Kruskal–Wallis and Mann–Whitney tests were used to evaluate pH, Colony Forming Unit (CFU) count and volumetric roughness (Sa).

## Results

The pH results are shown in Table 1. No statistically significant pH variations were found between the attachments of the excess group, scalpel and 24-blade bur group for the 24 h evaluation period. On the other hand, all groups differed statistically (*p* < 0.05) from the control group (specimens without biofilm). This group had a pH 7.4 and the other experimental groups ranged from 5.3 to 5.9.

**Table 1:** Kruskal–Wallis and Mann–Whitney test results and distribution of quantitative variables according to pH and CFU counts for the experimental groups.

Groups	pH				CFU			
	Mean	Min	Max	SD	Mean	Min	Max	SD
Excess	5.85 **A**	5.83	5.88	0.02	709.70 **A**	49.5	3416	221.8
Scalpel	5.85 **A**	5.84	5.88	0.01	342.65 **B**	85	900.5	84.8
Low-speed 24-blade	5.88 **A**	5.84	5.92	0.02	78.30 **C**	24.5	217	38.7
Control	7.46 **B**	7.42	7.48	0.02	–	–	–	–

*Mean* arithmetic mean, *Min* minimum, *Max* maximum, *SD* standard deviation. Different letters indicate statistical difference among groups (*p* < 0.05).

The direct macroscopic count of microorganisms on the plates can be seen in Table 1. There was a statistically significant difference between the mean number of CFUs: Excess (709.70 ± 221.8), Scalpel (342.65 ± 84.8) and 24-blade low-speed bur (78.3 ± 38.7), respectively. The Excess group had higher number of CFU (*p* < 0.05), while the low-speed 24- blade drill group had the lowest number of CFU (*p* < 0.05).

Regarding the analysis of non-contact 3D profilometry (Table 2), for the initial Sa, the 24-blade low-speed bur and Scalpel groups were statistically similar, and both differed from the Excess group (*p* < 0.05). For the final Sa, the samples from the 24-blade low-speed bur group showed the lowest roughness value, while the Scalpel group showed intermediate values, however they all differed statistically (*p* < 0.05). The Excess group was the most expressive in terms of final volumetric roughness.

**Table 2:** Kruskal–Wallis and Mann–Whitney test results and distribution of initial and final surface roughness (Sa, µm) for the experimental groups.

Groups	Initial Sa (µm)				Final Sa (µm)			
	Mean	Min	Max	SD	Mean	Min	Max	SD
Excess	190.45 **Aa**	43.83	401.20	145.33	120.61 **Aa**	30.51	42.88	83.62
Scalpel	70.16 **Bb**	50.37	256.89	85.10	41.34 **Bb**	40.62	167.35	53.65
Low-speed 24-blade	94.39 **Cc**	60.80	493.91	157.01	100.77 **Cc**	30.30	25.74	37.02

*Mean* arithmetic mean, *Min* minimum, *Max* maximum, *SD* standard deviation. Different letters indicate statistical difference among groups (*p* < 0.05).

**Fig. 2:**
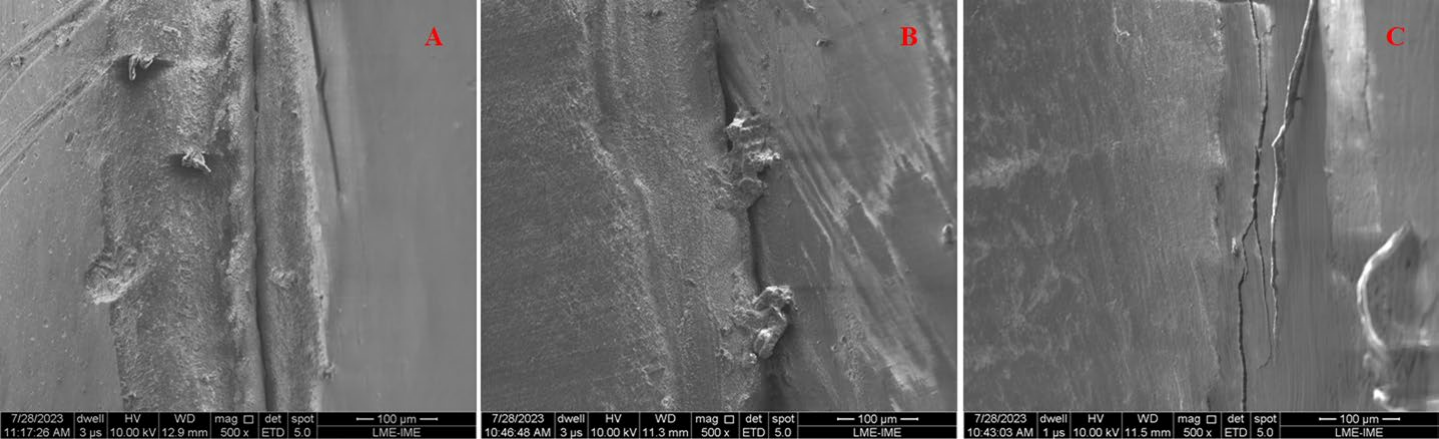
SEM images at 500× magnification showing the interface of 3 M Resin Z100 attachments subjected to different forms of excess removal. (**A**) no excess removal; (**B**) excesses removed by scalpel blade n° 15; (**C**) excesses removed by low-speed 24-blade drill.

More robust edges could be seen in attachments with excess, forming a valley aspect with the support base. When removed with a scalpel blade, the attachment’s edge has a ramp-like appearance, tapering from its body to the support surface. The edge of the attachment removed with 24-blade low-speed bur seems to be more perpendicular (Fig. 2).

## Discussion

The present study demonstrates that the method used for removal of excess composite resin significantly influences surface roughness, microbial adhesion, and biofilm acidogenicity on orthodontic attachments. These findings highlight that finishing is not merely a technical step, but a biologically relevant determinant of surface behavior in the oral environment.

Surface roughness is a well-established factor in bacterial colonization. In restorative dentistry, it has long been suggested that surfaces exceeding a threshold roughness of approximately 0.2 μm favor plaque retention^13^. In the present investigation, specimens with residual excess resin exhibited the highest roughness values and, correspondingly, the highest CFU counts. This supports the concept that micro- and nano-scale irregularities act as sheltered niches that protect bacteria from shear forces and facilitate early biofilm stabilization.

The superior performance of the 24-blade low-speed bur in reducing both surface roughness and microbial counts aligns with previous evidence showing that fine carbide burs or multi-step polishing systems produce smoother composite surfaces than incomplete or coarse finishing approaches^14^. Although that study evaluated restorative composites rather than orthodontic attachments, the underlying principle remains consistent: the quality of finishing directly determines surface morphology and, consequently, biofilm-related outcomes. Similarly, Jefferies et al*.* emphasized that instrument selection influences not only roughness but also long-term plaque accumulation potential^15^. The present results extend this restorative framework into orthodontics, where attachment finishing is routine yet insufficiently standardized.

Beyond roughness magnitude, surface morphology and marginal geometry appear clinically relevant. Attachments with residual excess resin displayed irregular, valley-like margins, which may function as stagnation sites. Marginal discrepancies and overhangs in restorative dentistry are known to increase localized plaque retention and gingival inflammation, reinforcing the importance of precise contouring in minimizing ecological shifts within the biofilm.

The microbiological findings further strengthen the biological interpretation. The Excess group not only demonstrated the highest CFU counts but also showed greater pH reduction, indicating a more acidogenic biofilm. Acidification of the local microenvironment is directly associated with enamel demineralization and white-spot lesion development during orthodontic treatment^16^. While that study focused on fixed appliances, the biological mechanism—plaque retention combined with acidogenic biofilm activity—remains applicable to composite attachment surfaces. In this context, smoother attachment surfaces may contribute not only to reduced bacterial accumulation but also to lower cariogenic potential.

The scalpel technique yielded intermediate roughness and microbial outcomes but showed greater variability, likely reflecting operator-dependent factors such as angulation, pressure, and tactile control. This variability underscores a broader issue: finishing procedures are inherently technique-sensitive. Even under standardized laboratory conditions, subtle procedural differences may influence final surface characteristics. In clinical practice, such variability may be amplified, reinforcing the need for clearer guidelines and potentially more reproducible finishing systems.

Several considerations must be acknowledged when interpreting these findings. This study was conducted using an in vitro biofilm model, which allows controlled evaluation of surface–microbe interactions but does not fully reproduce the complexity of the oral environment. Factors such as salivary flow, acquired pellicle dynamics, dietary challenges, and host immune responses were not simulated^17,18^. Additionally, only one composite resin type was evaluated. Differences in filler size, filler loading, resin matrix composition, and degree of conversion may influence how materials respond to finishing procedures and subsequent microbial colonization.

The incubation period represented early biofilm formation and may not reflect the behavior of mature, multispecies biofilms over extended timeframes. Furthermore, no thermocycling or mechanical wear simulation was performed. In vivo, orthodontic attachments are exposed to temperature fluctuations, masticatory forces, brushing abrasion, and continuous contact with thermoplastic aligners, all of which may alter surface roughness over time. Future investigations should therefore incorporate artificial aging protocols, longer biofilm maturation periods, and multispecies microbial models^19^. Comparative studies including multiple composite formulations, additional finishing and polishing systems, and wet versus dry finishing conditions would further clarify the clinical relevance of these findings. Quantitative assessment of surface wettability and surface free energy may also help elucidate the physicochemical mechanisms underlying bacterial adhesion^19,20^. Ultimately, longitudinal in vivo studies are necessary to validate whether improved finishing translates into reduced plaque accumulation and decreased enamel demineralization adjacent to aligner attachments.

Beyond microbial considerations, the potential biomechanical implications of finishing protocols should also be acknowledged. Aligner attachments are designed to generate specific force vectors and enhance mechanical retention between the aligner and the tooth surface. Their geometry, edge definition, and surface integrity are critical for predictable force transmission and aligner tracking. Excess resin or irregular finishing may alter attachment morphology, potentially compromising the precision of its designed contours. Conversely, overly aggressive rotary finishing could theoretically reduce attachment volume or modify critical edges, potentially affecting retention and the efficiency of force delivery. Although the present study did not directly evaluate attachment stability or aligner tracking, surface morphology differences observed between the scalpel and 24-blade bur groups suggest that finishing protocols may influence not only microbial adhesion but also the mechanical interaction between the attachment and the thermoplastic aligner. Future studies should therefore investigate whether different finishing approaches affect attachment wear, detachment rates, aligner seating accuracy, and overall treatment predictability in clinical settings.

Taken together, the present results indicate that finishing technique is not merely a procedural detail but a clinically meaningful determinant of surface topography, microbial colonization, and biofilm acidogenicity. The use of a low-speed 24-blade bur resulted in smoother surfaces, reduced bacterial adhesion, and lower acidogenic potential compared to scalpel finishing or residual excess resin. These findings support the development of more standardized, evidence-based protocols for attachment finishing in aligner therapy, with the aim of minimizing plaque retention and associated biological complications.

## Conclusion

Resin finishing protocols critically influence the surface roughness and biofilm behavior of orthodontic attachments. Excess resin produced the highest roughness and bacterial load, whereas the low-speed 24-blade bur generated the smoothest surfaces and minimized microbial accumulation and biofilm acidity. These findings underscore that meticulous finishing is essential to reduce plaque retention and may meaningfully lower the risk of enamel demineralization during orthodontic treatment.

## Data Availability

The datasets used and/or analysed during the current study available from the corresponding author on reasonable request.
